# Exploring facilitators and barriers to early TB case finding at private community pharmacies in Kampala, Uganda using the Consolidated Framework for Implementation Research (CFIR)

**DOI:** 10.21203/rs.3.rs-7196158/v1

**Published:** 2025-08-19

**Authors:** Rodgers Katwesigye, Mary Mbuliro, Rejani Lalitha, Richard Katuramu, Alfred Andama, Stavia Turyahabwe, Moorine Sekadde, Fred C. Semitala

**Affiliations:** Makerere University Joint AIDS Program (MJAP); Makerere University Joint AIDS Program (MJAP); Makerere University College of Health Sciences; Busitema University; Busitema University; Ministry of Health Uganda; Ministry of Health Uganda; Makerere University College of Health Sciences

**Keywords:** Tuberculosis, private community pharmacies, Healthcare providers

## Abstract

**Background:**

Tuberculosis (TB) remains one of the leading global infectious diseases killer, with Uganda among the countries bearing the highest TB/HIV burden. The World Health Organization’s (WHO) ambitious End TB strategy by 2030 emphasizes the strong involvement of private healthcare providers in TB efforts. In line with this, Uganda has adopted the WHO’s public-private mix (PPM) model. This study explored the facilitators and barriers to engaging private community pharmacies in the early detection of TB cases in Kampala, Uganda.

**Design/Methods:**

We conducted a qualitative study at five private community pharmacies in Kampala. We used in-depth interviews with healthcare providers (HCPs) dispensing medications at private community pharmacies, pharmacy clients, and key informant interviews with pharmacy owners/managers. Data was analyzed using an inductive thematic approach, identifying themes as barriers or facilitators to engaging private community pharmacies in TB case finding. These themes were then mapped to the Consolidated Framework for Implementation Research (CFIR) domains and constructs.

**Results:**

Facilitators of TB screening at private community pharmacies include: pharmacy staff’s willingness to be trained and collaborate with healthcare professionals to screen for TB. Healthcare providers acknowledge TB as a serious public health threat and view community pharmacies as valuable partners in early detection and prevention. Leveraging existing community awareness and targeted communication campaigns can further enhance patient engagement in TB screening services.

The barriers identified include limited space and the high facility expansion costs, inadequate access to TB screening tools and equipment, and persistent stigma and public misconceptions about TB that may deter patients from seeking screening. Pharmacy staff also face knowledge gaps, resource constraints, and potential revenue losses from referring patients to hospitals.

**Conclusions:**

These findings provide a basis for designing contextually appropriate interventions targeting factors that are likely to promote the engagement of private community pharmacies in Uganda in early TB case findings. Future studies should assess the impact of addressing identified barriers.

## Background

Tuberculosis (TB) remains the second leading cause of death from infectious diseases globally, following COVID-19, and ranks 13th among all causes of mortality worldwide[1]. It is also the leading cause of death among people living with HIV. In 2021 alone, TB claimed an estimated 1.6 million lives, including 187,000 individuals co-infected with HIV[1].

Uganda is among the 20 high-burden TB/HIV countries in the world, with a TB prevalence of 253 per 100,000 people and a higher prevalence in urban (504/100000) than rural (370/100000) settings[2].

In 2021, an estimated 91,000 people developed TB in Uganda, of whom 16,000 people were neither diagnosed nor notified [1]. This translates to 17.5% of TB cases remaining undetected and untreated in the community, posing a continued risk for ongoing transmission

The World Health Organization (WHO) global End TB strategy recommends a strong coalition and engagement of communities, civil society organizations, and public and private care providers to end TB by 2030[3].

Uganda adopted the WHO public-private mix (PPM) model to facilitate the involvement of private healthcare providers, including private community pharmacies, in TB care[4]. However, limited evidence exists to inform the development of a comprehensive policy on Public-Private Partnerships (PPP) for TB case detection. Strengthening private sector engagement—particularly through private community pharmacies—is a key priority in Uganda’s National TB and Leprosy Program (NTLP) Strategic Plan 2020–2025 and the National Health Sector Development Plan II[5].

Uganda adopted the WHO-recommended Public-Private Mix (PPM) model to facilitate the involvement of private healthcare providers, including community pharmacies, in TB care.

Several studies suggest that the success of this strategy will depend, in part, on effectively engaging retail pharmacies in TB screening and referral of presumptive TB cases, which is critical for accelerating the early detection and treatment of missing TB cases [6], [7].

**Figure 1:**
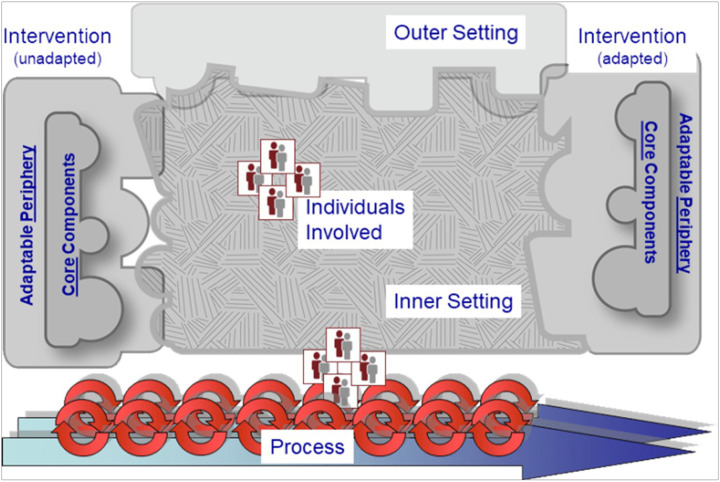
Illustration of the CFIR adapted from https://cfirguide.org/cfirdiagram/

However, the extent to which private community pharmacies contribute to TB case detection remains unclear despite their role as a first common point of contact for individuals with presumptive TB. This study explored the perspectives of both healthcare providers and recipients of care on TB screening at private community pharmacies in Kampala, with the aim of informing strategies to enhance early TB case finding. We applied the Consolidated Framework for Implementation Research (CFIR), as illustrated in Fig. 1, to examine the barriers to and facilitators of TB screening at community pharmacies. CFIR was selected because it offers a pragmatic and comprehensive structure for identifying multi-level factors that influence implementation. CFIR is particularly useful in diverse settings, enabling researchers to assess what works, for whom, and under what conditions-especially in complex health systems such as those in low-and middle-income countries [8].

CFIR synthesizes constructs from multiple theories and disciplines into a unified framework, organized across five interrelated domains that collectively influence implementation processes and outcomes. The updated CFIR 2.0 includes the following domains: Innovation, Outer Setting, Inner Setting, Individuals, and Process[9]. These domains interact dynamically, allowing for a nuanced understanding of context-specific challenges and opportunities for implementation.

## Study design

In February 2023, we conducted in-depth interviews with healthcare providers (HCPs) responsible for dispensing medications in community pharmacies and in-depth interviews with clients presenting at community pharmacies. These interviews took place at five purposively selected community pharmacies in Kampala, Uganda’s capital.

Additionally, we conducted key informant interviews with owners/managers of private community pharmacies to generate broader perspectives on how to engage private community pharmacies in Kampala in early TB case finding.

We used the Consolidated Criteria for Reporting Qualitative Research (COREQ) guidelines in reporting this qualitative study (see Additional file 1). The study was approved by the AIDS Support Organization (TASO) Research and Ethics Committee **(TASO REC 010/2021-UG-REC-009)** and the Uganda National Council of Science and Technology **(SS804ES)**.

### Study setting

This study was conducted at five private community pharmacies in Kampala, the capital city of Uganda. Kampala hosts the highest concentration of community pharmacies in the country and contributes a substantial proportion of Uganda’s annual TB case load[10], [11].

We purposively selected five pharmacies situated in high-density, low-income areas of the city, including vicinities near major markets, taxi parks, and informal settlements (slums). These areas were identified as potential TB transmission hotspots due to high-density population and pervasive poverty, both well-recognized risk factors for TB. The selection aimed to capture settings where community pharmacy-based TB screening interventions could yield the highest public health impact.

### Participant selection and recruitment

We purposively selected healthcare providers (HCPs) involved in medication dispensing and clients who sought services at the participating community pharmacies. Participant recruitment was guided by the principle of data saturation as outlined by Lincoln and Guba[12], who advocate for a naturalistic inquiry approach rooted in studying phenomena within their real-world contexts using purposive sampling and inductive analysis.

In line with ethical considerations and respect for participant privacy, HCPs and clients were approached in person at the pharmacy premises. We provided a clear explanation of the study’s purpose, emphasizing that their perspectives would contribute to the development of a contextually appropriate strategy for engaging private community pharmacies in early TB case detection.

### Study instruments and data collection

We developed semi-structured interview guides (see Additional Files 2, 3, and 4) with open-ended questions tailored to explore perceived barriers and facilitators to engaging private community pharmacies in early TB case finding in Kampala. These guides were originally drafted in English; pilot tested with healthcare providers and clients at a community pharmacy not involved in the study and refined.

Interviews lasted between 25 and 40 minutes and were conducted with healthcare providers, pharmacy clients, and key informants. All participants provided written informed consent prior to participation. Interviews were audio-recorded with permission and transcribed verbatim. Transcripts were anonymized and stored in a secure, password-protected digital folder accessible only to the research team.

### Research team

All Interviews were conducted in community pharmacy settings by a multidisciplinary team that included implementation scientists, social scientists, physicians, and national TB program personnel. The data collection team received training prior to fieldwork and was supervised throughout the process. Initial interviews were carried out by a trained social scientist with support from a note-taker. Interviewers had no prior relationship with study participants.

## Data analysis

Data were analyzed thematically by a team of five researchers (FCS, RK, ST, RL, and AA). We preferred thematic analysis because it is suitable for examining the perspectives of different research participants, highlighting similarities and differences, and generating unanticipated insights[13]. We adopted an inductive approach using open coding that facilitated the identification of themes within the data. Initially, two analysts (FCS, RK) read three similar transcripts independently, familiarizing themselves with the data and documenting thoughts on potential codes and themes. Thereafter, the transcriptions were exported to Atlas. ti version 8. The team collaboratively developed and refined a coding framework and met regularly to reconcile differences and reach consensus on key themes.

We categorized the emerging themes as either facilitators or barriers to TB screening in community pharmacies, depending on whether they supported or hindered the implementation of screening efforts.

We extracted specific quotations from the transcripts to illustrate verbatim expressions of matters that appeared important.

We developed a behavioral diagnosis by mapping the emergent facilitators and barriers onto their associated CFIR domains and constructs.

## Results

### Demographic characteristics of study participants

The study included 20 participants comprising three groups: healthcare providers (n = 8), pharmacy managers (n = 2), and clients of community pharmacies (n = 10). Among the healthcare providers, six were female and two were male; five were nurses and three were pharmacy technicians. Both pharmacy managers were female nurses. Of the ten clients, six were female and four were male.

### Potential facilitators for TB screening at private community pharmacies

#### Pharmacy staff’s willingness to learn and collaborate with experts

Healthcare providers at the pharmacy expressed eagerness to receive more training and improve their knowledge of TB screening, which can facilitate successful implementation.

“Training would be good… We need knowledge about screening TB”

(R-10)

“I would prefer to consult with experts around Mulago (National Referral Hospital) and have them available for advice.”

(R-09)

#### Public health needs, as TB is a public health threat

Healthcare providers reported that they already appreciate TB as a public health threat and feel that community pharmacies can contribute to TB screening as a valuable public health service, especially in areas where early detection and management can prevent disease spread.

“We already know that TB is there in the communities, and we get clients with persistent coughs. Pharmacy TB screening will help in early detection of TB in individuals with persistent coughs.”

(R-01)

#### Existing community awareness and communication

Some healthcare providers reported that they already conduct regular awareness and communication with their clients through posters displayed in the pharmacy and one-on-one interactions. They suggested that TB screening services can also be added to these activities to boost engagement and raise awareness about TB screening and encourage clients of community pharmacies to seek screening.

“We already conduct health education through posters and one-on-one discussions with our clients. We would require TB screening posters for the clients; it is one of the easiest ways of communicating.”

(R-06)

### Potential barriers for TB screening at private community pharmacies

#### Space constraints

The lack of designated private areas for screening within the community pharmacies limits the ability to conduct TB screening and collect sputum samples effectively.

“The screening area, I don’t think I have anywhere to screen from. So that makes it hard. So, will I tell the client to sit there and wait? How will they give me their sputum?”

(R-05)

#### Increased operational costs

The financial implications of TB screening services were also a significant concern. Pharmacy managers reported that adding TB screening services may result in extra costs for space expansion, equipment, and additional staff.

“There is a need to allocate a private room for screening, requiring additional space and partitioning the pharmacy, which could lead to extra costs.”

(R-08)

#### Stigma around TB

Healthcare providers and clients of community pharmacies noted that stigma around TB could prevent pharmacy clients from engaging in TB screening services, especially in community settings.

“Some clients may not return or might not prioritize the test even after encouraging and advising them to get screened for TB.”

(R-07)

#### Knowledge gaps among healthcare providers

Healthcare providers reported having insufficient skills and limited knowledge of TB screening and treatment, which might hinder the effective delivery of TB screening services in community pharmacies.

“We are not specialized in handling TB cases… We need knowledge about TB screening and treatment.”

(R-01)

#### Public perception and misconceptions about TB

Healthcare providers reported that the clients they received, and the pharmacies, may be reluctant to undergo TB screening due to misconceptions about TB, the screening process, or a lack of knowledge about its symptoms.

“It’s difficult to convince people to take a TB test, and there is fear and resistance among people when they hear about TB testing and screening.”

(R-03)

#### Lack of tools for TB screening and sputum collection tools

Healthcare providers reported lacking access to necessary TB screening tools and equipment to carry out the screening.

“We need tools and equipment for TB screening to conduct the tests.”

(R-01)

#### Negative impact on revenue

There is concern that TB screening might reduce pharmacy revenue as clients may be lost to hospital referrals before purchasing medications.

“Implementing TB screening may affect revenue as some clients might be lost to screening before purchasing medicines.”

(R-03)

#### Need for incentives and motivation

Healthcare providers reported that they require additional motivation to conduct TB screening at community pharmacies. They reported that offering financial incentives or other benefits could encourage staff to engage more fully in TB screening:

“Providing monetary incentives or job security serves as motivation for healthcare workers to engage in TB screening.”

(R-01)

#### Mapping onto the CFIR

Table 1 below provides a summary of the facilitators and barriers to engaging private community pharmacies in TB screening mapped onto the CFIR.

## Discussion

Engaging private community pharmacies in TB case finding presents a valuable opportunity to address the gaps in early detection and management of TB in Uganda. This study explored the facilitators and barriers to engaging private community pharmacies in early tuberculosis (TB) case finding in Kampala, Uganda, using the Consolidated Framework for Implementation Research (CFIR). Our findings align with global efforts to enhance public-private partnerships in TB care and highlight critical factors influencing the integration of TB screening services in private pharmacies[4].

Our findings echo previous studies in low- and middle-income countries (LMICs) that emphasize the critical role of private pharmacies in early TB case detection. For instance, studies conducted in India, Nepal, and Pakistan have demonstrated the feasibility of engaging private pharmacies in TB care, particularly in screening and referral processes, when combined with appropriate training and incentives[6], [14], [15], [16], [17], [18]. Similar to our findings, these studies identified a lack of knowledge and resources among pharmacy staff as key barriers to implementation.

Additionally, the importance of addressing stigma and public misconceptions about TB has been highlighted in multiple contexts, where community-based interventions have successfully reduced stigma and increased patient uptake of TB screening[19]. These findings suggest that targeted community awareness campaigns, such as those proposed in our study, are critical to fostering acceptance and trust in pharmacy-based TB screening programs.

### Facilitators to TB screening

Several facilitators emerged from our study that are consistent with global implementation strategies. The willingness of pharmacy staff to undergo training reflects a positive attitude toward professional development and public health engagement. Similar findings have been reported in studies, where training programs significantly enhanced the capacity and confidence of pharmacy staff to perform TB screening and referrals[17].

Collaboration with healthcare experts, particularly from public health institutions, was identified as a valuable strategy for bridging knowledge gaps and supporting implementation efforts. Previous research has demonstrated the effectiveness of such collaborations in improving the quality and consistency of TB referrals from private pharmacies[18].

### Barriers to TB screening

Despite these opportunities, significant barriers remain. Space limitations and the associated costs of expanding facilities, procuring equipment, and hiring additional staff were frequently cited as challenges. Similar resource constraints have been reported in studies where infrastructure inadequacies hindered the scale-up of pharmacy-based TB services[20].

Stigma and misconceptions about TB remain pervasive barriers, deterring patients from seeking care. This is consistent with findings from studies in similar settings, where interventions to normalize TB screening through community engagement and education successfully improved uptake[21].

Resource limitations, particularly concerns about revenue loss due to hospital referrals, were another significant challenge identified in our study. This finding aligns with reports from similar settings, where pharmacies expressed similar apprehensions about financial losses associated with referral-based TB care models[22].

### Implications for policy and practice

To address these barriers, policymakers and stakeholders should prioritize implementation strategies such as creating linkages between the community pharmacies and public health facilities to facilitate sample collection, testing, diagnosis, and referral. Providing financial and material incentives to pharmacy staff can help offset implementation costs and enhance motivation, as demonstrated in successful pharmacy-based TB screening programs [17]. Investments in training programs to equip staff with the skills and knowledge needed for TB screening are equally critical.

Community engagement strategies that reduce stigma and normalize TB screening are essential for improving patient uptake. For example, integrating TB awareness campaigns with existing health promotion activities has proven effective in similar settings[23].

Lastly, designing implementation strategies that support infrastructure development, such as subsidizing equipment and providing portable diagnostic tools, can help overcome space and resource constraints.

### Strengths and limitations

This study is among the first to systematically explore the barriers and facilitators to engaging private pharmacies in TB case finding in Uganda using the CFIR framework. The qualitative approach provided rich insights into stakeholder perspectives, offering a nuanced understanding of implementation challenges.

However, the purposive selection of pharmacies and participants may limit the generalizability of findings to other settings. Additionally, the reliance on self-reported data introduces the possibility of bias, particularly regarding sensitive issues like stigma and financial concerns. Future studies should incorporate quantitative assessments and pilot interventions to validate these findings and assess their broader applicability.

## Conclusions

Engaging private community pharmacies in TB case finding is feasible and presents a valuable opportunity to address the gap in early detection and management of TB in Uganda. By addressing the identified barriers and leveraging the facilitators, stakeholders can design effective, contextually tailored interventions that enhance TB screening.

## Supplementary Material

Supplementary Files

This is a list of supplementary files associated with this preprint. Click to download.

- COREQCHECKLIST.docx
- IDIguideprivatecommunitypharmacyclients.docx
- KIIguidewithprivatecommunitypharmacyowners.docx
- IDIguidewithprivatecommunitypharmacyhealthcareproviders.docx

## Figures and Tables

**Table 1:** CFIR Mapping of Facilitators and Barriers for TB Screening at Community Pharmacies

CFIR Domain	CFIR Construct	Facilitator	Barrier
Innovation	Innovation Cost		Increased operational costs
Individuals	Innovation Deliverers	Pharmacy staff’s willingness to learn and collaborate with healthcare professionals	
	Characteristics sub domain (Capability)		Knowledge gaps among pharmacy staff regarding TB screening
Outer Setting	Local Attitudes	Recognition of TB as a public health threat	
	Local Attitudes		Stigma and public misconceptions about TB
	Partnerships & Connections		Potential revenue loss from hospital referrals
Inner Setting	Communications	Existing community awareness and communication	
	Structural Characteristics (Physical infrastructure)		Space constraints
	Structural Characteristics (Physical infrastructure)		Lack of screening tools and sputum collection equipment
	Incentive Systems		Need for incentives and motivation

Abbreviations: CFIR – Consolidated framework for implementation research

## Data Availability

The datasets used and/or analyzed during the current study are available from the corresponding author on reasonable request.
